# Supplementary material for: Ultrafast electron dynamics at the Dirac node of the topological insulator Sb2Te3
Source: Sci Rep. 2015 Aug 21;5:13213. doi: 10.1038/srep13213 (PMC4543953; doi:10.1038/srep13213)
Supplement: Supplementary Information [file srep13213-s3.pdf]

Supplementary Information for: Ultrafast electron dynamics at the Dirac node of the topological insulator Sb_2Te_3

Siyuan Zhu[1,*], Yukiaki Ishida[2], Kenta Kuroda[1], Kazuki Sumida[1], Mao Ye[3], Jiajia Wang[4], Hong Pan[4], Masaki Taniguchi[1], Shan Qiao[3,4], Shik Shin[2] & Akio Kimura[1]

[1] *Graduate School of Science, Hiroshima University, 1-3-1 Kagamiyama, Higashi-Hiroshima, Hiroshima 739-8526, Japan*

[2] *Institute for Solid State Physics, the University of Tokyo, 5-1-5, Kashiwa-no-ha, Chiba 277-8581, Japan*

[3] *State Key Laboratory of Functional Materials for Informatics, Shanghai, Institute of Microsystem and Information Technology, Chinese Academy of Sciences, 865 Chang Ning Road, Shanghai 200050, China*

[4] *Department of Physics, State Key Laboratory of Surface Physics, and Laboratory of Advanced Materials, Fudan University, Shanghai 200433, China*

I Comparison of bulk and surface electron dynamics

To compare the electron dynamics of bulk and surface states, we altered the pump and probe delay and investigated the time dependent variations in the TrARPES images. Fig. S1(a) shows the difference image along the $\overline{\Gamma}-\overline{K}$ line measured at $t = 0.26$ ps. Here the bulk conduction band and two branches of UDC are represented as Bulk, Surface_ A and Surface_ B, respectively. We set energy and momentum frames and plotted the normalized intensity variation in each frame as a function of t [see Fig. S1(b)-(i)]. The intensities in the same energy range are normalized. The intensity variations of bulk and surface states do not show any significant differences. That tells us that the bulk and surface electron dynamics only depend on the energy.

II Simulation of decay behavior from thermal distribution

Here we show how the excited electrons decay via electronic temperature cooling. First of all, we show the Fermi Dirac distribution function with a temperature $T = T_0$ at the 'zero' delay time (t=0), as shown in the inset panel of Fig. S2. Then we assume that T exponentially decays as described with $T = T_1 + (T_0 - T_1) * exp(-t/\tau)$, where

T_0, T_1 denote the initial and the equilibrium temperatures, and τ expresses the decay rate. We plot the electron occupation probabilities at five binding energies $E1$, $E2$, $E3$, $E4$ and $E5$ (marked in the inset panel) as shown in Fig. S2. We can find that the decay rate increases as the energy approaches the Fermi energy. The result of this simulation shows that the thermal decay cannot explain the observed population inversion.

III Simulation of population inversion

To qualitatively demonstrate the population inversion, we draw the decay lines for the uniform DOS with the non-uniform one. Here we consider a simple model with 10 energy windows, marked as S0-S9. We assume that the electron transfer takes place only between the adjacent windows. The binding energy S9 is the highest and that of S0 is the lowest. The intensity of S0 is assumed to be caused only by a direct excitation. The intensity of S9 is considered to follow an exponential decay.

Considering a uniform DOS, as shown in the upper panel of Fig. S3 (c), we can simulate the decay lines of different windows. As shown in Fig. S3 (a), the high binding energy window shows the earlier rising edge Therefore the uniform DOS cannot be the origin of the population inversion.

The non-uniform DOS, where the DOS in the region S7 is reduced down to to 10% of the others is assumed for the simulation as shown in the lower panel of Fig. S3 (c). Here, all the other parameters are set to the same values as those used for the uniform DOS. As shown in Fig. S3 (b), a big change occurs for the regions S6-S8, while the higher energy regions do not change significantly. The intensity of S8 shifts to earlier delay time than that of S6, just like what we experimentally observed for UDC and LDC. This result can reasonably explain that the observed population inversion takes place due to the bottleneck effect near Dirac node.

Figure S1: Decay behaviors of bulk and surface electrons (a) The difference image at $t = 0.26$ ps. The bulk conduction band and two branches of UDC are marked as Bulk, Surface_ A and Surface_ B, respectively. (b)-(i)The decay behaviour of Bulk, Surface_ A and Surface_ B at different energy ranges (340-360 meV, 360-380 meV, 380-400 meV, 400-420 meV, 420-440 meV, 440-460 meV, 460-480 meV and 480-500 meV).

Figure S2: Simulation of decay behavior from thermal distribution The intensity variation lines at different binding energy E1, E2, E3, E4 and E5, as marked in the inset panel, which shows the thermal distribution at a delay time of 0ps.

Figure S3: Simulation of a simple model for hourglass effect (a) Simulated decay lines assuming a uniform DOS distribution. (b) Simulated decay lines assuming 10% DOS at energy region S7. (c) Schematic figures for the assumed DOS distributions in the simulation.

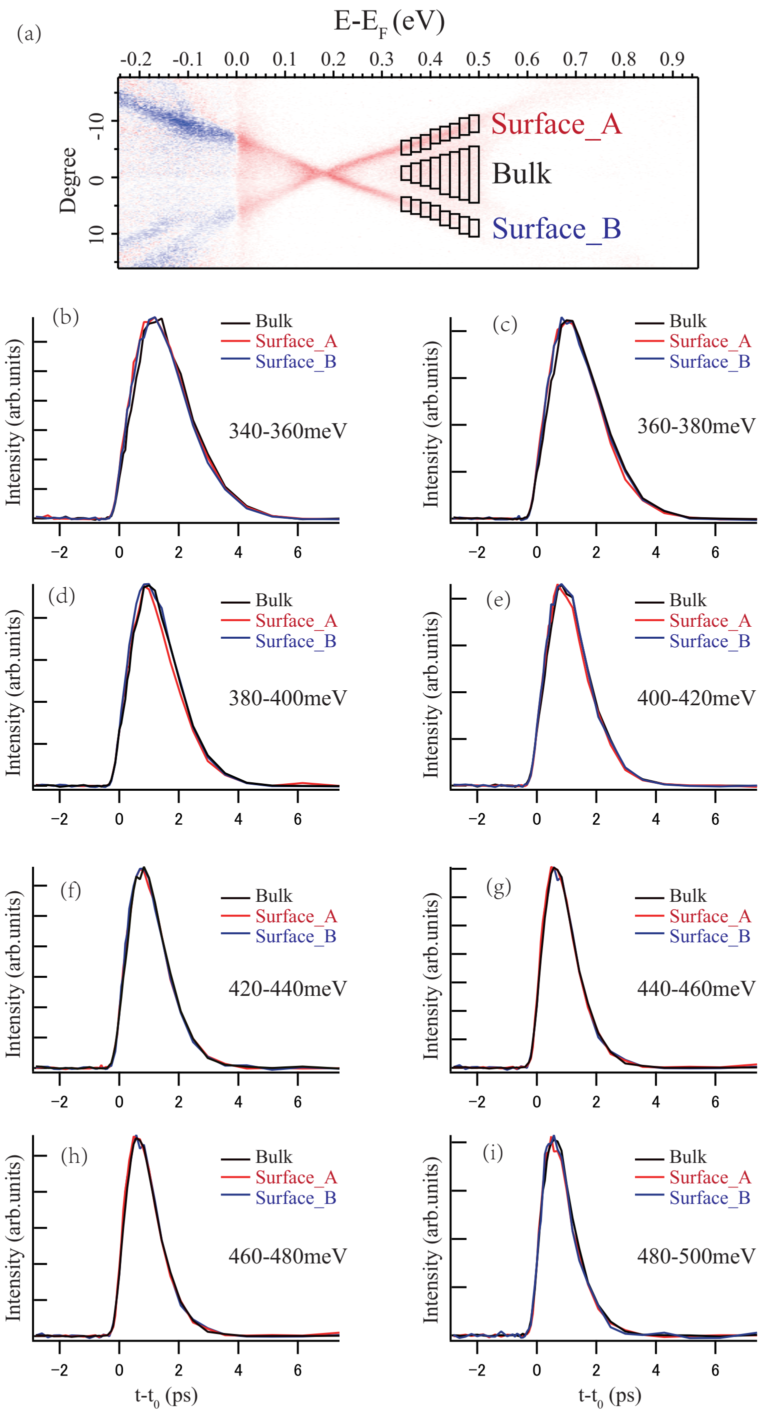

(a)
E-E_F (eV)
-0.2 -0.1 0.0 0.1 0.2 0.3 0.4 0.5 0.6 0.7 0.8 0.9
Degree
-10
0
10
Surface_A
Bulk
Surface_B
(b)
(c)
(d)
(e)
(f)
(g)
(h)
(i)
Bulk
Surface_A
Surface_B
340-360meV
360-380meV
380-400meV
400-420meV
420-440meV
440-460meV
460-480meV
480-500meV
Intensity (arb.units)
−2 0 2 4 6
t-t_0 (ps)

Figure 1:

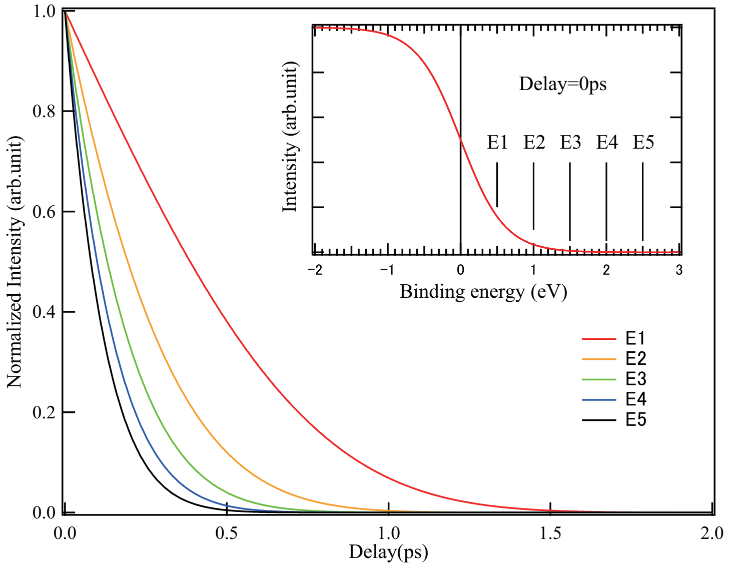

Figure 2:

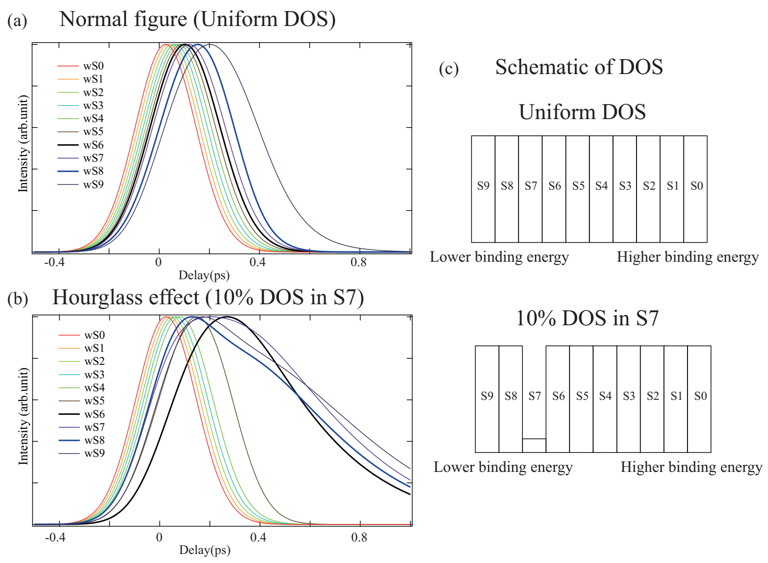

Figure 3:
